# Dynamics of digestive vacuole differentiation clarified by the observation of living *Paramecium bursaria*

**DOI:** 10.1007/s00709-024-01996-1

**Published:** 2024-10-08

**Authors:** Keiko Obayashi, Yuuki Kodama

**Affiliations:** 1 Major in Agricultural and Life Sciences, Graduate School of Natural Science and Technology, Shimane University, Matsue-Shi, Japan; 2 Institute of Agricultural and Life Sciences, Academic Assembly, Shimane University, Matsue-Shi, Japan

**Keywords:** *Chlorella* sp., Corning^®^ Cell-Tak™, Digestive process, Digestive vacuole, Endosymbiosis, *Paramecium bursaria*

## Abstract

*Paramecium bursaria* is a ciliate species that has a symbiotic relationship with *Chlorella* spp. This study aimed to elucidate the dynamics of digestive vacuole (DV) differentiation in *P. bursaria*, using yeast stained with a pH indicator. Previously, DV differentiation in *P. bursaria* has been classified into eight periods based on fixed-cell observations. However, to understand the behavior and physiology of *P. bursaria* in its natural state, it is essential to observe living cells. This study presented a novel method using Cornig® Cell-Tak™ to immobilize living *P. bursaria* cells, which enabled long-term observation of the same cell from the same direction. This technique allowed for real-time observation of DV differentiation, including the relationship between changes in the internal pH of DV and the diameter of DV, yeast budding from the DV membrane by a single cell into the cytoplasm, and separation of a DV containing multiple yeasts into two DVs. This study provides new insights into the dynamic process of DV differentiation in *P. bursaria*. These findings contribute to a better understanding of the cellular mechanisms underlying the symbiotic relationship between the two organisms and shed light on the complex process of intracellular digestion in ciliates.

**Supplementary Information:**

The online version contains supplementary material available at 10.1007/s00709-024-01996-1.

## Introduction

*Paramecium* is a common unicellular organism widespread in freshwater. The cell surface of *Paramecium* is covered with numerous cilia, which can move around in water by creating water currents. The cytopharynx of ciliates is densely covered with cilia, and by moving them, the surrounding bacteria, algae, and other particles are stirred up and taken into the cell. Substances taken into the cell through the cytopharynx are encased in digestive vacuoles (DV)s or phagosomes. Phagocytosis and intracellular digestion have been studied in detail in the genus *Paramecium* (Allen and Fok 2000). The DV of *Paramecium* sp. begins to form when the discoidal vesicle membrane fuses with the membrane of the cytopharynx and detaches from the cytopharynx to form DV. The newly formed nascent DV is referred to as the DV-I. Discoidal vesicle membranes are transported from the cytoplasm to the cytopharyngeal area along the rails of microtubules. After acidosomes that cause acidification of the DVs are fused, the pH inside the DV is approximately 3. This acidified DV is referred to as the DV-II. The acidosomal membrane is then recovered as budding, and lysosomes containing digestive enzymes are fused; this DV is termed DV-III. Eventually, the lysosomal membrane is recovered by budding, and this DV is called DV-IV. Finally, DV-IV adheres to the microtubules present near the cytoproct and fuses with the cell membrane of the cytoproct to eject undigested material in the DV to the extracellular space.

*Paramecium bursaria*, a species of the genus *Paramecium*, is characterized by a symbiosis of approximately 700 *Chlorella* sp. in its cytoplasm (Kodama and Fujishima 2008). The host *P. bursaria* supplies the symbiotic *Chlorella* spp. with nitrogen sources and carbon dioxide (Reisser 1976, 1980; Albers et al. 1982; Albers and Wiessner 1985); the algae supply the host with the photosynthetic products, oxygen, and sugars mainly maltose (Muscatine et al. 1967; Brown and Nielsen 1974; Reisser 1986). Thus, the relationship between *P. bursaria* and *Chlorella* spp. is mutualistic. However, both organisms can multiply on their own independently. Therefore, it is possible to produce algae-removed *P. bursaria* cells (henceforth referred to as alga-free *P. bursaria* cells) in a non-symbiotic state, in which symbiotic algae are artificially removed. When symbiotic *Chlorella* spp. isolated from algae-bearing *P. bursaria* cells and alga-free *P. bursaria* cells are mixed, some of the algae incorporated into DVs escape digestion and resume endosymbiosis (Siegel and Karakashian 1959; Karakashian 1975). Re-endosymbiosis experiments between alga-free *P. bursaria* and *Chlorella* sp. were conducted, and differentiation of the DVs of *P. bursaria* was clarified. The differentiation process of DVs had been classified into eight periods (Kodama and Fujishima 2005). When alga-free *P. bursaria* was mixed with the isolated symbiotic *Chlorella* sp., the algae were taken up through the cytopharynx and enclosed in DV-I. The DV-I membrane was visible under a light microscope and observed within 30 s after mixing with the algae. The intravacuolar pH is 6.4–7.0. Acidified and condensed DV-II by the acidosomal fusion to DV-I appeared at 0.5–1 min after mixing. The DV-II membrane was difficult to observe under a light microscope and the intravacuolar pH rapidly decreased to 2.4–3.0. Lysosome fusion occurred at 2–3 min, the DV membrane expanded and differentiated into DV-III, and the membrane was observable again under a light microscope. The intravacuolar pH increases to 6.4–7.0. DV-III can be divided into three types: DV-IIIa, in which all algae in its DV are undigested and green; DV-IIIb, in which yellow-faded-digested algae and green algae coexist because digestion has begun; and DV-IIIc, in which all algae are yellow-faded-digested. After 20 min, the DV membrane differentiated into DV-IV and contracted again. DV-IV also can be divided into three types: DV-IVa, in which all algae in its DV are still undigested and green; DV-IVb, in which brown-digested algae and green algae coexist; and DV-IVc, in which all the algae inside were digested and the color was brown. During the differentiation process of these DVs, it has been shown that most algae that succeed in endosymbiosis emerge from DV-IVb 30 min after mixing with alga-free *P. bursaria* by budding of the DV membrane enclosing single green alga (Kodama et al. 2007). In this way, the DVs of *P. bursaria* undergo dynamic changes during algal re-endosymbiosis process, including changes in intravacuolar pH and budding from the DV membrane into the cytoplasm. However, these studies were conducted using *P. bursaria* cells fixed with paraformaldehyde, and there is no method for the long-term observation of living cells. The component of Corning® Cell-Tak™ (Cell-Tak) is a specially formulated protein extracted from the marine mussel, *Mytilus eduli*s (Instructions for Use, https://www.scientificlabs.co.uk/handlers/libraryFiles.ashx?filename=Manuals_3_354240_A.pdf). This protein is designed to be used as a coating on a substrate to immobilize cells or tissues but has never been used in *Paramecium* spp. to our knowledge. In this study, to clarify the DV differentiation process of *P. bursaria* using live cells, yeast stained with a pH indicator dye was fed to alga-free *P. bursaria*, which were attached to glass slides using Cell-Tak. Then, we observed the yeast-enclosing DVs of living *P. bursaria *for a long time.

## Material and methods

### Cultivation of *Paramecium* spp.

*Paramecium multimicronucleatum* strain YM-25 (syngen 2, mating type III or IV), *P. tetraurelia* strain 51 (mating type E), and alga-free *P. bursaria* strain Yad1w (syngen R3, mating type III) were used in this study. Strain Yad1w was produced from algae-bearing *P. bursaria* strain Yad1g (Kodama and Fujishima 2009a). All *Paramecium* spp. were cultured using red pea (*Pisum sativum*) extract culture medium (Tsukii et al. 1995) in Dryl’s solution (NaH_2_PO_4_·2H_2_O replaced with KH_2_PO_4_) (Dryl 1959) and inoculated with non-pathogenic *Klebsiella aerogenes* (ATCC35028) as food bacteria 1 d before use (Fujishima et al. 1990). For ordinary cultures, several hundred *Paramecium* spp. cells were inoculated 2 ml aliquots of the culture medium in glass test tubes. Subsequently, 2 ml of fresh culture medium was added daily for 12 days. Cultures in the early stationary phase, 1 day after the last feeding were used in all experiments.

### Preparation of glass slides and coverslips coated with Corning® Cell-Tak™

**Fig. 1:**
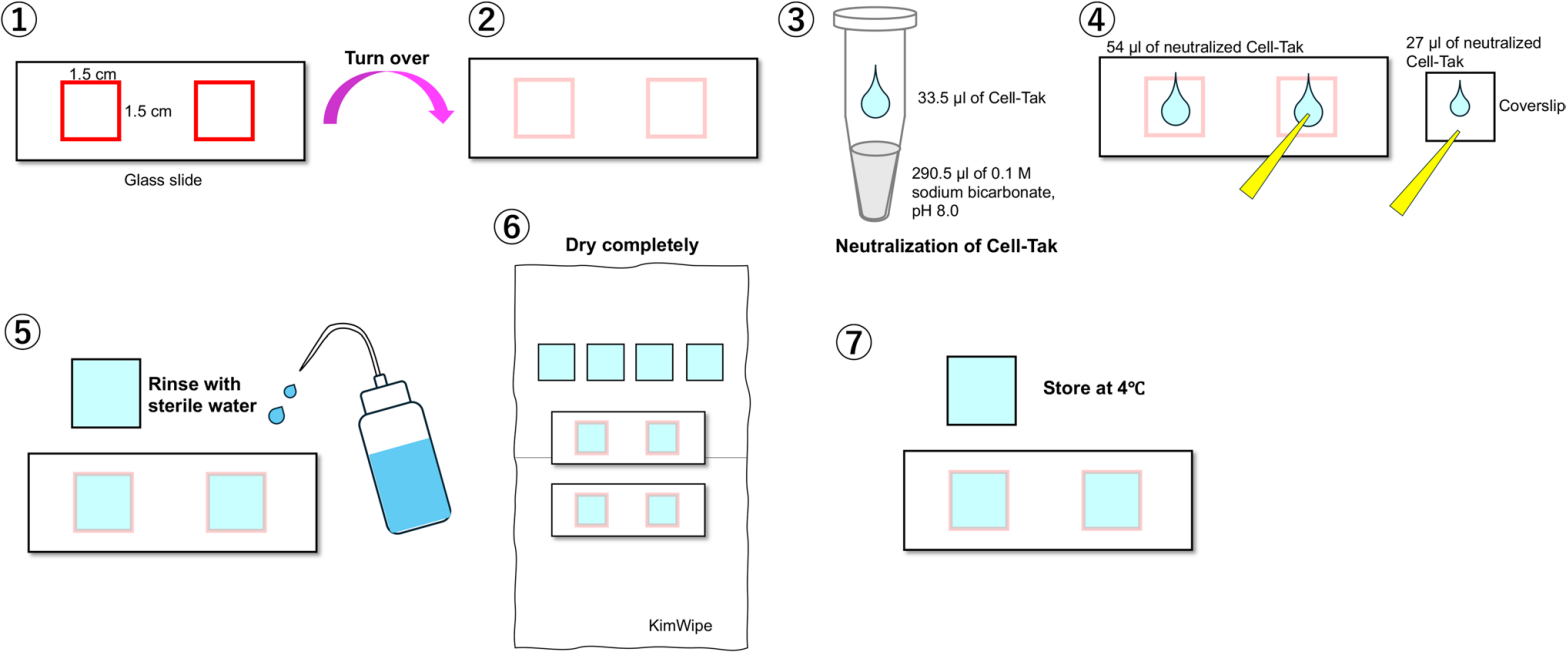
Methods for preparing glass slides and cover slips coated with Cell-Tak. See the “Materials and methods” section for further details

Glass slides and coverslips were coated with Corning® Cell-Tak™ (Cell-Tak) according to the manufacturer’s instructions. The adsorption method was used as the coating method, and we adjusted to approximately 4.0 μg Cell-Tak per cm^2^, as recommended in the instructions. Two 15 mm × 15 mm square frames were drawn on a 76 mm × 26 mm glass slide, with a red oil-based magic marker spaced at least 5 mm apart. The glass slide was then turned over with nothing written side facing up. Subsequent procedures were performed on a clean bench. For Cell-Tak neutralization, 33.5 µl of Cell-Tak (formulation 1.78 mg/ml in 5% acetic acid) and 290.5 µl of 0.1 M sodium bicarbonate solution (pH 8.0) were mixed in a 1.5-ml tube. For each square of the glass slide (2.25 cm^2^), 54 µl of diluted neutralized Cell-Tak was placed on the glass slide. Half of the volume for the glass slide (27 µl) was placed on a coverslip. The diluted neutralized Cell-Tak was spread onto the glass with a micropipette tip (200 µl). The spread process was performed within 5 min of adjusting the neutralized Cell-Tak, because adsorption begins immediately after neutralization. The glasses were incubated on a clean bench for at least 20 min after the application of the neutralized Cell-Tak. The glass slides and coverslips were rinsed with sterile water to remove residual sodium bicarbonate and then placed on Kimwipes. After they were completely dried and stored at 4 °C until use, the glasses were used within 14 days. The procedure is illustrated in Fig. 1.

### Adhesion of *Paramecium* spp. with Cell-Tak

All *Paramecium* spp. cells from a 180 to 240 ml culture in the early stationary phase of growth were strained through two layers of Kimwipes to remove gross debris. The cells were transferred to a plastic beaker equipped with nylon mesh with a pore size of 15 μm. All *Paramecium* spp. cells were harvested, and the cell suspension was washed by pouring 100 ml of Dryl’s solution into a plastic beaker and concentrated to 1 ml. The cell density was adjusted to 10,000 cells/ml. A 14 µl drop of the cell suspension was placed on a Cell-Tak–coated glass slide, and a coverslip coated with Cell-Tak was placed on it. The slides were immediately observed under a differential interference contrast (DIC) microscope (BX53; Evident, Tokyo, Japan), and cells that did not migrate on the slides were counted as adhered *Paramecium* spp. cells.

### Yeast cells stained with pH indicator dye, Congo Red

*Saccharomyces cerevisiae* strain BY611 was provided by the National Bio-Resource Project (NBRP), Japan. BY611 cells stained with the pH indicator dye Congo Red were prepared as described previously (Kodama and Fujishima 2005). Yeast cells were cultivated on ordinary agar plates (Miyakawa et al. 1984) and suspended in Dryl’s solution. A 100 μl aliquot of yeast suspension and 1 ml of 0.5% Congo Red dissolved in distilled water were mixed and incubated at 100 °C for 60 min. The mixture was centrifuged at 4500 × g for 1 min at 23 ± 1 °C, washed five times with Dryl’s solution under the same centrifugation conditions, stored at − 20 °C, and used for experiments within 1 month of staining. Yeast cell density was calculated using a Thoma blood-counting chamber.

**Fig. 2:**
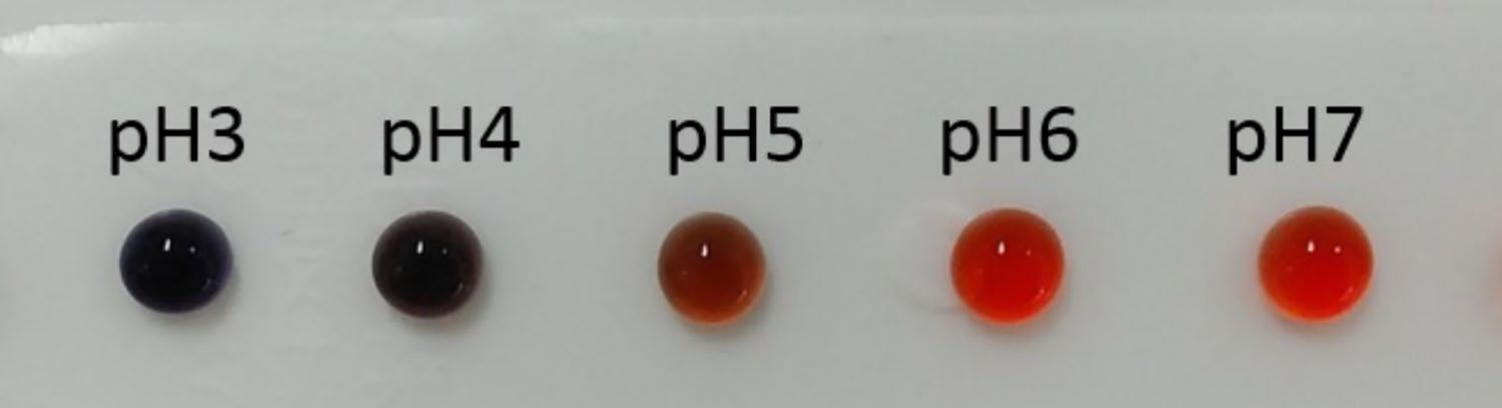
The color change of Congo Red at each pH value was confirmed using 100 mM citrate buffer. Congo Red is blue at pH 3, violet at pH 4, purplish-red at pH 5, and red at pH 6 and 7

Congo Red is blue at pH 3 or lower and red at pH 5 or higher. The color change of Congo Red with pH was confirmed using 100 mM citric acid buffer at pH 3–7 (Fig. 2).

### Mixing experiment of stained yeast and alga-free *P. bursaria* cells

Alga-free *P. bursaria* cells were prepared as previously described. Alga-free *P. bursaria* cells and stained yeast were mixed at 5 × 10^3^ *P. bursaria* cells/ml and 5 × 10^7^ yeast cells/ml, respectively, at 23 ± 1 °C in a 10 ml centrifuge tube for 1.5 min. Then, undigested yeast cells were separated from *P. bursaria *cells by using a nylon mesh with a pore size of 15 μm and pouring 100 ml of Dryl’s solution. The paramecia retained in the mesh were transferred to a new 10-ml centrifuge tube and resuspended in Dryl’s solution. When appropriate, 14–18 µl of *P. bursaria* cells from a 10-ml centrifuge tube were placed on a Cell-Tak–coated glass slide, as described above, and observed using a DIC microscope. Dryl’s solution was added with a micropipette as to prevent the prepared slide from drying out during observation, appropriately. Observation was stopped if adhesion was dislocated, release of trichocysts due to drying or pressure stress, or a decrease in the speed of cytoplasmic streaming was observed. Photomicrographs and video images were captured with a DP74 microscope (Evident, Tokyo, Japan). The mean diameter of the DVs was measured using the Olympus cellSens Dimension software (Evident, Tokyo, Japan).

### Quantification and statistical analysis

Mann–Whitney *U* test and two-sided Fisher’s exact test were used for the statistical evaluation of the results. Statistical significance was set at *P* < 0.05. All data were presented as mean ± standard deviation (SD). All statistical analyses were performed using R software (R Ver 4.1.3) (Ihaka and Gentleman 1996).

## Results and discussion

### Attachment of *Paramecium* spp. by Cell-Tak

**Fig. 3:**
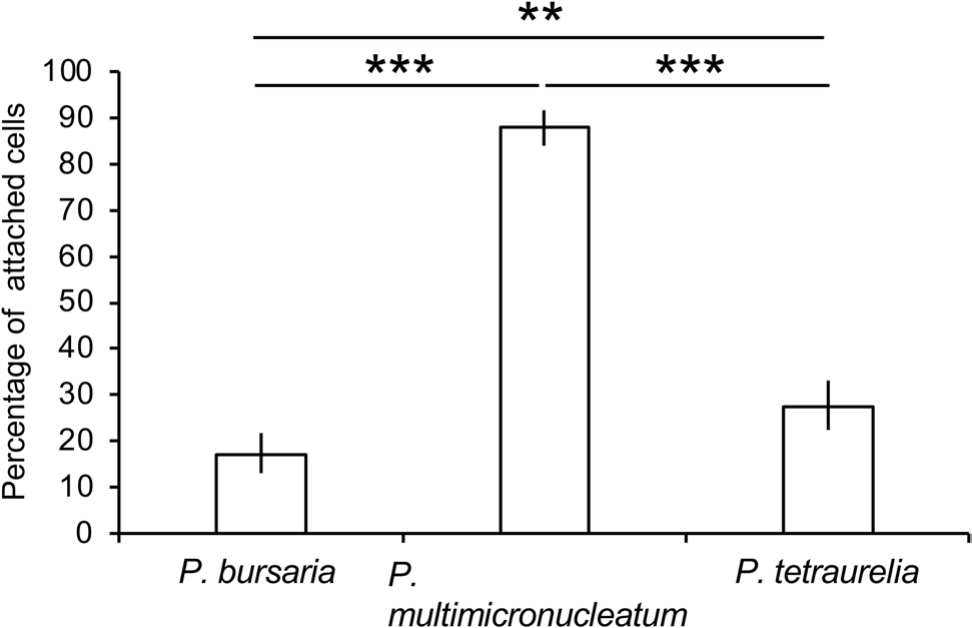
Comparison of ease of adhesion of *Paramecium* spp. cells to glass slides coated with Cell-Tak. In *P. bursaria*, *P. multimicronucleatum*, and *P. tetraurelia*, 17%, 88.1%, and 27.6% of the cells, respectively, were attached. ** and *** mean statistical differences with Fisher’s exact test, **P, 0.01 and ***P, 0.001. *P. bursaria* exhibited the weakest adhesion among the three species of *Paramecium* spp. examined

The percentage of cells adhering to the glass slides coated with Cell-Tak is shown in Fig. 3. *P. bursaria*, 17% (50 cells/294 observed cells), *P. multimicronucleatum*, 88.1% (259 cells/294 observed cells), and *P. tetraurelia*, 27.6% (50 cells/294 observed cells) were attached. The Fisher's exact test showed significant differences between *P. bursaria* and *P. multimicronucleatum*, *P. multimicronucleatum* and *P. tetraurelia* (*P* < 0.001), and *P. bursaria* and *P. tetraurelia* (*P* < 0.01). In other words, *P. bursaria* was the least adhesive of the three *Paramecium* spp. The reproducibility of this experiment was confirmed three times.

*Paramecium multimicronucleatum* had the longest duration of continuous adhesion (> 4 h), whereas *P. bursaria* and *P. tetraurelia* had the longest durations (< 2 h). The cells of the three species differed in size, with *P. multimicronucleatum* being the largest and *P. bursaria* being the smallest. Because cells were trapped between the glass slide and coverslip, the ease of adhesion may be related to the size of the area in contact with Cell-Tak. The most likely site of adhesion between *Paramecium* spp. cells and Cell-Tak is the cilia on the outer surface of the cells; however, this could not be confirmed under a DIC microscope. Strong shaking in a solution containing 5% ethanol can quickly removed cilia, except for those in the cytopharynx of *Paramecium* (Ogura 1981). Observations using cilia-removed cells may confirm the ease of adhesion and involvement of cilia.

Because *Paramecium* spp. move around actively through ciliary movements, it is difficult to observe them without fixation. Therefore, a common method of observing *Paramecium* spp. in vivo is to observe them after treatment to weaken their locomotion. Methods to weaken movement include increasing the viscosity of the external fluid with 2–5% methylcellulose to physically prevent swimming and treating the cells with chemicals such as 4 mM nickel chloride solution to inhibit ciliary movement (Shigenaka 1988). However, even with these methods, it is not possible to completely stop cell swimming, and it is difficult to observe over a long period of time. The observation of living *Paramecium* spp. cells is crucial for understanding their behavior and physiology in the natural state. Although adhesion rates and adhesion times vary among different species of the genus *Paramecium*, observations using Cell-Tak have the advantage of allowing prolonged observation of live cells, which have high swimming ability, from one direction.

### Observation of DVs of alga-free *P. bursaria* ingesting Congo Red-stained yeast

**Fig. 4:**
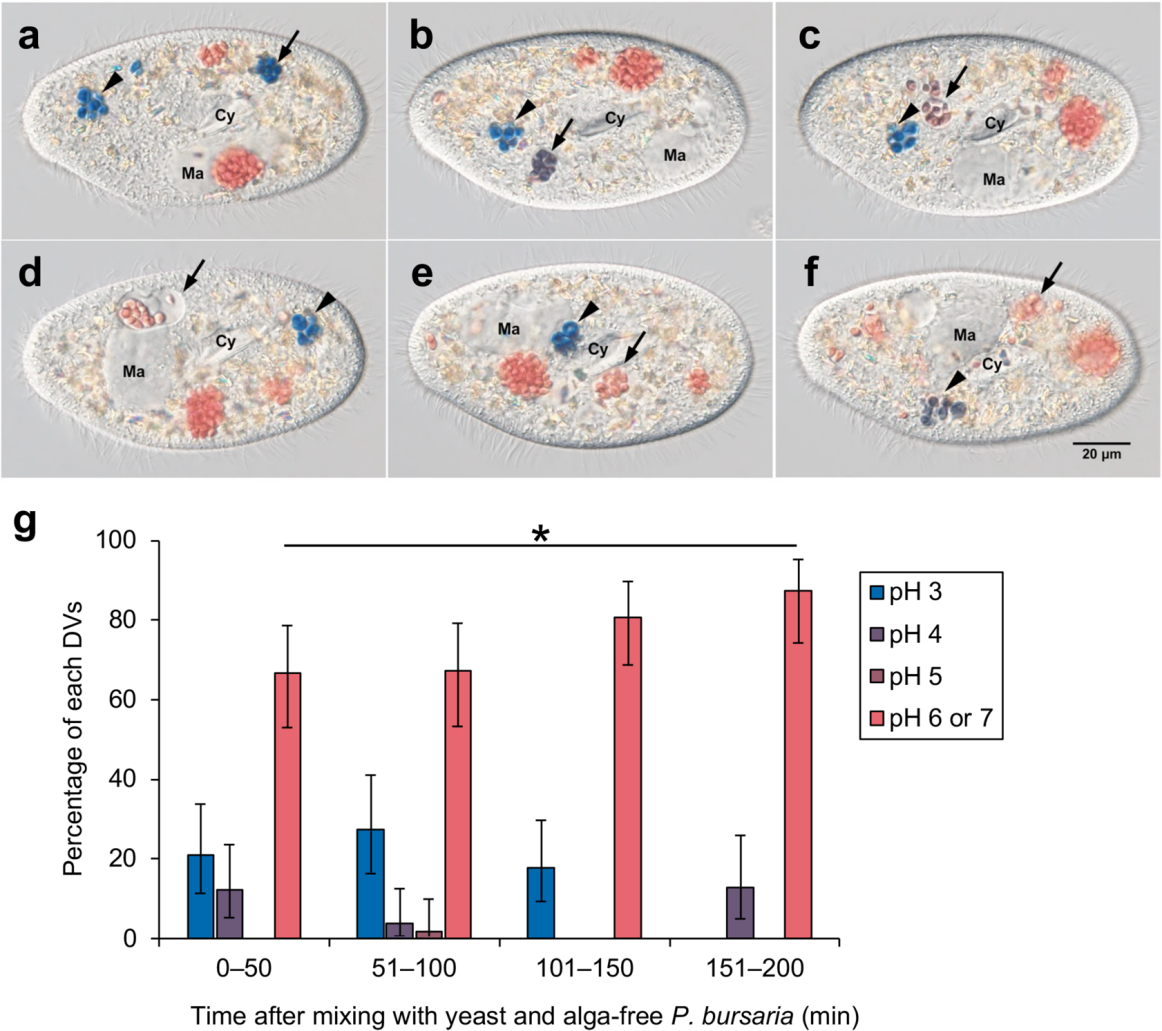
Changes in pH of DVs during phagocytosis observed over time. The same cell was able to be observed from 1 h 6 min 53 s to 2 h 58 min 46 s after mixing. (**a**–**f**) Photomicrographs of alga-free *P. bursaria* cells with yeast observed at 1 h 7 min 44 s (**a**), 1 h 20 min 17 s (**b**), 1 h 21 min 48 s (**c**), 1 h 27 min 4 s (**d**), 2 h 6 min 53 s (**e**), and 2 h 44 min 10 s (**f**). The color of the yeast in the DV, indicated by the arrow, changed from blue to purple, reddish-purple, and red over time. Therefore, it is presumed that the pH in DV increased stepwise from approximately 3 to 4 and from approximately 5 to 6. The color of yeast in the DV, indicated by the arrowhead, changed from blue to purple over time. Therefore, it was presumed that the pH of the vesicles increased from approximately 3 to 4. Ma, macronucleus; Cy, cytopharynx. **g** shows the changes in the internal pH of the DVs over time. The number of acidic DVs decreased over time, and many of the DVs were neutral. * means statistical differences with Mann–Whitney’s *U* test, *P, 0.05

**Fig. 5:**
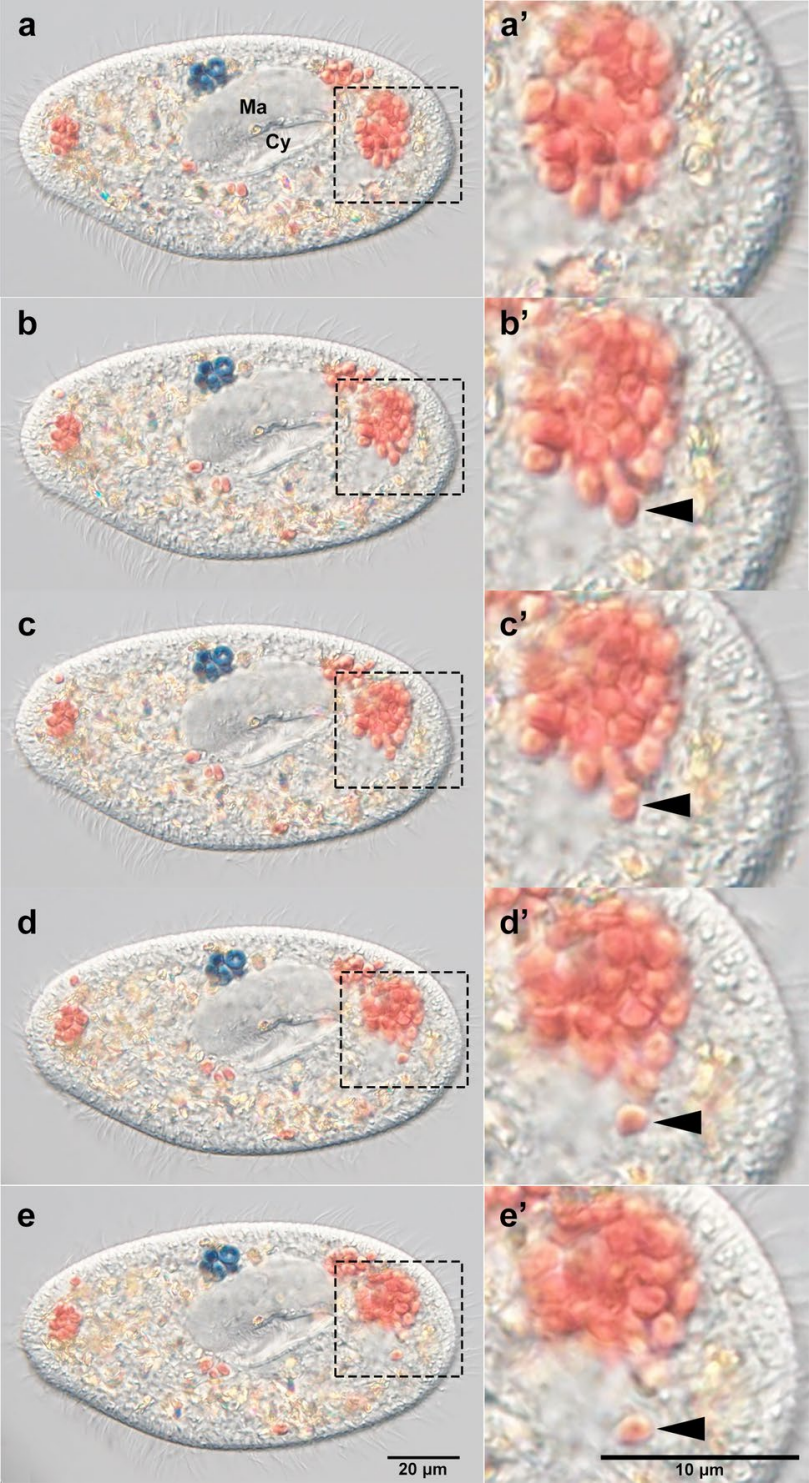
Moment when the yeast in the DV bud by a single cell one into the cytoplasm of alga-free *P. bursaria*. (**a**–**e**) Photomicrographs of alga-free *P. bursaria* cells with yeast observed at 2 h 29 min 59 s (**a**), 2 h 30 min 2 s (**b**), 2 h 30 min 3 s (**c**), 2 h 30 min 6 s (**d**), and 2 h 30 min 7 s (**e**). (**a’**–**e’**) An enlargement of the square area depicted in (**a**–**e**). The time required for release of yeast cell singly from the DV, indicated by the arrow, was 8 s. Ma, macronucleus; Cy, cytopharynx

**Fig. 6:**
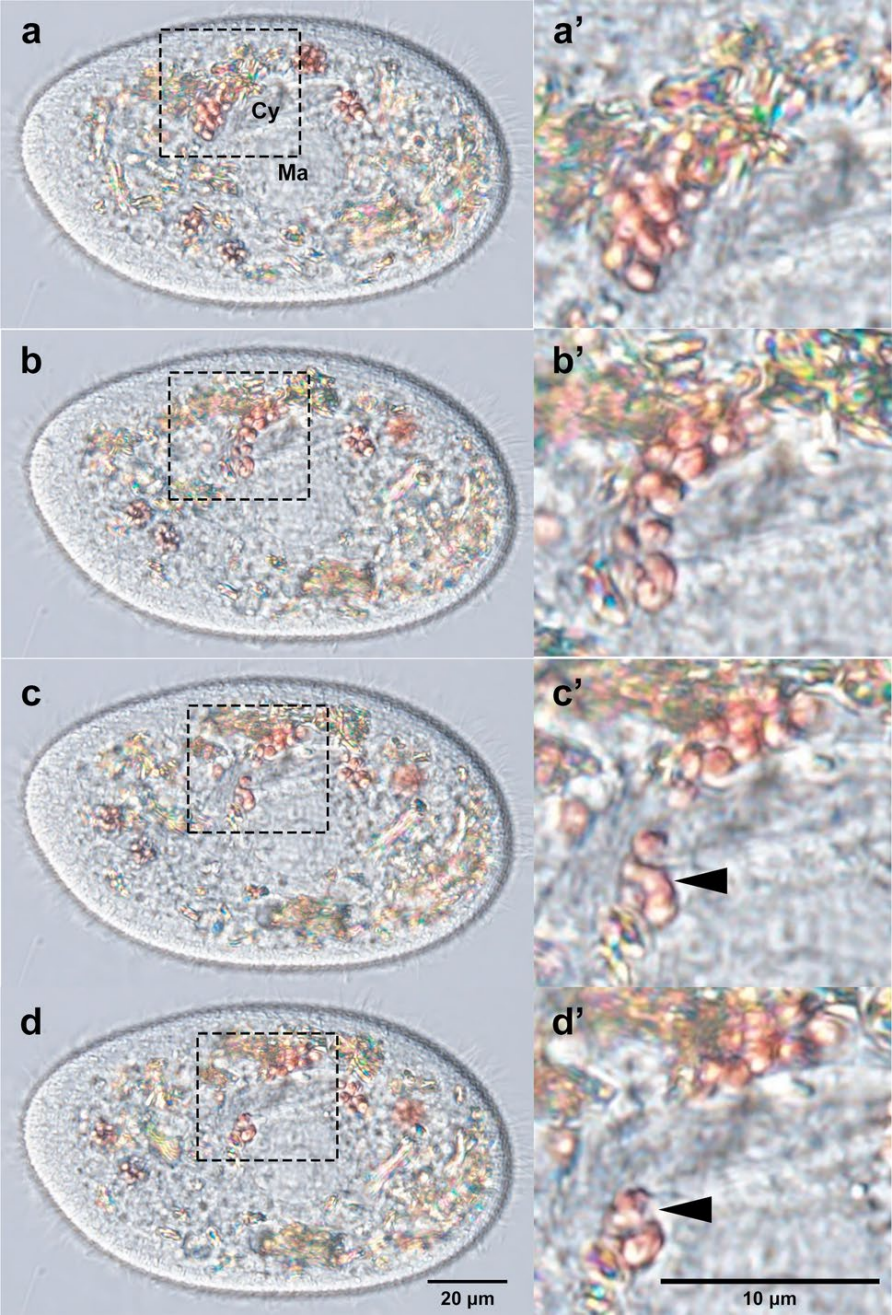
Moments when one DV is divided into two. The cell was observed from 5 min 37 s to 17 min 24 s after mixing. After mixing with yeast cells, alga-free *P. bursaria* cells were observed at 7 min 11 s (**a**), 7 min 22 s (**b**), 7 min 26 s (**c**), and 7 min 27 s (**d**). (**a’**–**d’**) An enlargement of the square area depicted in (**a**–**d**). As indicated by the arrowhead, multiple yeast-containing DV was separated into two DVs. Ma, macronucleus; Cy, cytopharynx

**Fig. 7:**
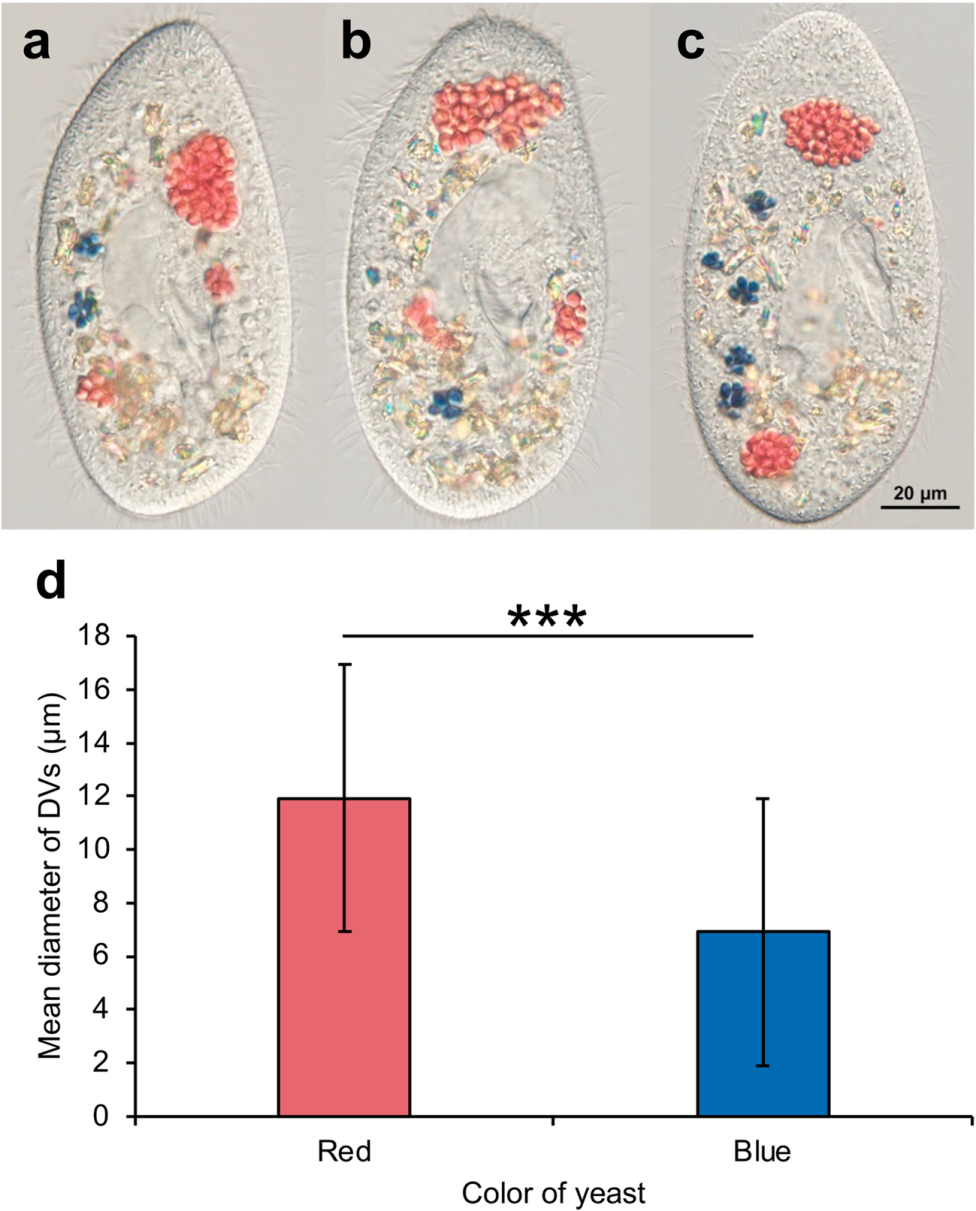
Relationship between the diameter of the DVs and the color of the yeast inside. (**a**) DIC image of a DV observed 16 min 36 s, (**b**) 28 min 44 s, and (**c**) 36 min 18 s after mixing with yeast. It can be seen that the yeast in the small-diameter DV is blue in color. Thus, the pH of these vacuoles was estimated to be 3. On the other hand, yeast a larger-diameter DV is red in color. Thus, the pH of these vacuoles was estimated as 6 or 7. (**d**) shows the relationship between the mean diameter of the DVs and the color of the yeast inside. The mean diameter of the DVs containing red yeast was 11.9 μm, whereas that of the DVs containing blue yeast was 6.9 μm. *** means statistical differences with Mann–Whitney’s *U* test, ***P, 0.001. Thus, it was suggested that the larger-diameter DVs are unlikely to acidify or are not sufficiently acidified

DIC microscopy images of living alga-free *P. bursaria* mixed with yeast stained with 0.5% Congo red are shown in Figs. 4, 5, 6 and 7.

Figure 4 shows the change in pH inside the DVs over time. The same cell was able to be observed from 1 h 7 min 44 s after mixing with yeast (a) to 2 h 44 min 10 s after mixing (f). In a–f, the DVs indicated by the arrows and arrowheads are the same. At 1 h 7 min 44 s after mixing, four relatively large DVs enclosing multiple yeasts were observed, of which the color of the yeast inside two of the DVs was red and the other two were blue (a). The DV membrane containing blue yeast, indicated by the arrow, gradually swelled over time, and the color of the yeast interior changed from blue to purple (b), then to red–purple (c), and then to red (d − f).The greatly swollen DV membrane (d) gradually contracted and adhered to the inside yeast, and the DV membrane was no longer observed under a DIC microscope (f). At 2 h 44 min 10 s after mixing (f), the yeast in the other DV, indicated by the arrowhead, also changed from blue (a–e) to purple (f). The yeast cells in the remaining two DVs, not indicated by arrows or arrowheads, showed no color change and remained red throughout the observation period. (g) demonstrates the temporal changes in the internal pH of the DVs. This result was obtained by observing 221 DVs. This graph illustrated that the proportion of acidic DVs decreased over time, with the majority of DVs ultimately showed a neutral pH. As shown in the “Introduction” section, DVs acidified by acidosomal fusion rapidly return to neutral (Kodama and Fujishima 2005). In this experiment, prolonged observation of the cells was feasible, revealing that acidification persisted in certain DVs even after a considerable duration following DV formation. However, the underlying mechanisms and reasons for maintenance of acidification remain unclear.

Figure 5 and Video S1 show that a single yeast cell from the DV membrane was released into the cytoplasm of the alga-free *P. bursaria*. The same cell was observed from 2 h 29 min 59 s after mixing (a) to 2 h 30 min 7 s after mixing (f). In (a), four relatively large DVs containing multiple yeast cells are observed, and yeast release was observed from the DV with the largest diameter, as indicated by the dashed square. Of the yeasts packed within the DV membrane in (a), indicated by the dashed square (a’), one cell budded into the cytoplasm of the alga-free *P. bursaria* in (b and b', arrowhead). The DV membrane that contained budded yeast swelled, and the yeasts within the DV were slightly loose (b'). The single yeast indicated by arrowheads was further budded at (c and c’, arrowhead), completely released from the DV membrane at (d and d’, arrowhead), and was then observed to initiate flow alone by *P. bursaria* cytoplasmic streaming at (e and e’, arrowhead). Since it took only 8 s for a single yeast to bud from the DV membrane, this is a moment that could only be captured by observation in a live cell. As mentioned in the “Introduction” section, in *P. bursaria*, it has been reported that *Chlorella* sp., which leads to successful endosymbiosis, emerges from DV-IVb 30 min after DV formation (Kodama and Fujishima 2005). In this study, yeast was also observed to be released from DV in a single cell, as shown in Fig. 5. Thus, we reconfirmed that in *P. bursaria*, contents in the late DV were budded off with a single cell, even if its contents are not symbiotic algae. In this study, we succeeded for the first time in observing the moment of budding of contents from DV using live *P. bursaria* cells. It is known that *P. bursaria* can recognize the size of contents in their DVs and induce budding of the DV membrane to establish endosymbiosis. Microbeads larger than 3.00 μm induced budding similar to symbiotic algae, whereas smaller microbeads did not. Dynasore, a dynamin inhibitor, greatly inhibits DV budding, suggesting that dynamin is involved in the budding process (Kodama and Fujishima 2012). In the case of *Chlorella* sp., which is capable of endosymbiosis with *P. bursaria*, it releases from the DV membrane into the cytoplasm one cell at a time and subsequently adheres beneath the host cell cortex to establish an endosymbiotic relationship (Kodama and Fujishima 2009a). In the present study, a single yeast cell budding from the DV membrane did not adhere to the cell cortex and was expelled after several days. If we observe the behavior of the symbiotic *Chlorella* sp. taken up into the DVs using Cell-Tak, we may be able to observe the moment of algal adhesion to the cell cortex.

Figure 6 and Video S2 show the moment when a portion of a DV containing multiple yeast cells was torn off and separated into two DVs. The same cell was observed from 7 min 11 s (a) after mixing to 7 min 27 s (d). In (a), five small-diameter DVs containing multiple red yeast cells were observed, of which the DV indicated by the dashed squares was separated into two. The glowing structures observed in *P. bursaria* are crystals that are more abundant in alga-free cells than in algae-bearing cells (Kodama et al. 2024). The DV membrane was difficult to observe in (a) because it overlapped with crystals (a’) and extended vertically in (b) and the yeast in the DV was transferred vertically (b’). In (b), the DV hit the cytopharynx of *P. bursaria* (Cy in a) on its way through the cell in cytoplasmic streaming, which triggered its tearing into two pieces (c’, arrowhead). See Video S2 for this moment. Subsequently, in (d), the separated DV is further away from the original DV (d’, arrowhead). These observations indicated that the DV of *P. bursaria* is subdivided intracellularly during phagocytosis. The subdivision mechanism of DVs is unknown. A similar phenomenon is the nipping-off of early DVs in the cytopharynx during DV formation, which involves actin. Cytochalasin B, a potent inhibitor of actin polymerization, inhibits the formation of nascent DV from the cytopharynx (Allen and Fok 1985; Fok et al. 1985). Cytochalasin B and latrunculin B, which are inhibitors of actin polymerization, have been found to completely inhibit the parturition of nascent DV in alga-free *P. bursaria* (Kodama, unpublished data).

Figure 7 shows the relationship between the diameter of the DV and the color of the yeast inside the DV. The diameter of the DV was proportional to the number of yeast cells in it. (a) DIC image of DVs observed 16 min 36 s after mixing with yeast, (b) 28 min 44 s, and (c) 36 min 18 s. a, b, and c represent different cells. Five DVs are clearly observed in (a). Yeast in small-diameter DVs with low uptake showed a blue color, while yeast in large-diameter DVs with high uptake showed a red color. Four DVs can be clearly observed in (b). The yeast in the larger-diameter DVs was also red. Seven DVs can be clearly observed in (c). Of the seven, the yeast within the five small-diameter DVs were blue. The color of the yeast in the large DVs did not change when observed for up to 1 h 49 min after mixing. (d) shows the relationship between the mean diameter of the DVs and the color of the yeast inside. DVs formed between 20 and 40 min after mixing with yeast were analyzed. The mean diameter of the DVs containing red yeast was 11.9 μm, whereas that of blue yeast was 6.9 μm. The Mann–Whitney *U* test results showed that they were significantly different (*P* < 0.001). In other words, DVs with large diameters do not acidify, or, if they do, they do not acidify sufficiently.

In *P. bursaria*, it has been reported that the larger the number of *Chlorella* sp. in a DV and the larger the diameter of the DV, the less easily internal algae are digested (Kodama and Fujishima 2007). The cause of the difficulty in digesting *Chlorella* sp. in large DVs is not yet clear, but it has been reported that acidification occurs faster in small DVs than in large DVs that incorporate large amounts of *Chlorella* sp. (Kodama et al. 2017). Furthermore, all DVs incorporating *Chlorella* sp., regardless of diameter, became positive for lysosomal acid phosphatase (AcPase) activity within 30 min of mixing (Kodama and Fujishima 2009b). It has also been shown that in *Paramecium* sp., inhibition of acidosome-DV fusion or a reduction in both the acidification rate and vacuolar-pH drop would inhibit lysosome-DV fusion (Fok et al. 1987). Our study revealed that yeast cells incorporated into large DVs were not fully acidified and remained neutral (Fig. 7). These findings suggest that, in *P. bursaria*, even though acidosomes fuse with large DVs, the pH inside the DVs does not drop below 3, suggesting that sufficient acidification may not have occurred. Table 1 summarizes the results of these previous studies and the findings of the present study. Acidosomal fusion is initiated, and the pH of the DV decreases from 7 to approximately 3 within 5 min (Fok et al. 1982). This drastic change in pH is responsible for killing food organisms and denaturing their proteins (Fok and Allen 1990).

**Table 1:** Relationship between DV diameter and various phenomena in DV

Phenomenon	Large DV	Small DV	Reference
Number of contents	Many	Few	Kodama et al. (2007) This study
Number of digested algae	Few	Many	Kodama et al. (2007)
Degree of acidification	Weak or not occur	Strong	This study
Presence of acid phosphatase activity	Occur	Occur	Kodama and Fujishima (2009b)

Phagosomes of paramecia, amoebae, and endosomes of fibroblasts and other mammalian cells are acidified before lysosomal fusion. Phagosomes containing *Legionella pneumophila* (Horwitz and Maxfield 1984), *Glugea hertwigi* spores (Sibley et al. 1985), and *Toxoplasma gondii* (Sibley et al. 1985) inhibit phagosomal acidification and subsequent lysosomal fusion. In the case of *Yersinia pseudotuberculosis*, live *Y. pseudotuberculosis* inhibited phagosomal acidification, and the pH within phagosomes containing live *Y. pseudotuberculosis* remained at approximately 6, whereas the pH within phagosomes containing dead *Y. pseudotuberculosis* fell to approximately 4.5 (Tsukano et al. 1999). Thus, it is very likely that lysosome-phagosome fusion and subsequent degradation cannot occur efficiently unless phagosomes are adequately acidified. Some intraphagosomal pathogens may be able to circumvent the cell’s normal digestive process, primarily by inhibiting the acidification step (Fok et al. 1987).

Karakashian and Karakashian (1973) found that the digestion of dead boiled algae was delayed when they were enclosed in the same DV as live algae, which is a clear indication that live algae can influence the digestive processes of the host (Karakashian and Karakashian 1973). This influence may be due to the prevention or delay of acidification and lysosomal fusion of the DVs. We found that when symbiotic algae isolated from algae-bearing paramecia were maintained under constant dark (DD) conditions for 24 h before mixing with alga-free paramecia, almost all algae were digested in the host DVs (Kodama and Fujishima 2014). This finding suggests that some unknown factors produced in response to light are prerequisites for algal resistance to the host lysosomal enzymes (Kodama and Fujishima 2014). Furthermore, we examined whether the symbiotic *Chlorella* sp. of *P. bursaria* could influence host digestion. As a result, acidification and lysosomal fusion occurred later in DVs containing living algae than in those containing boiled algae or latex spheres. These results suggest that some unknown factors in algae that are produced in response to light may be associated with alterations in the host digestive process and indicate that living algae can influence host digestive processes during the early stage of algal infection (Kodama et al. 2017).

## Conclusion

This study aimed to clarify the behavior of DVs in living *P. bursaria* cells. We developed a method to restrict the ciliary movement of *P. bursaria* and to facilitate intracellular observation. Alga-free *P. bursaria,* which ingested yeast stained with a pH indicator dye were observed by attaching them to a slide glass using a Cell-Tak. The observation method using Cell-Tak allowed long-term observation of the same cell from the same direction without pretreatment. This study showed that the DVs of *P. bursaria* do not cause sufficient acidification in the DV when the content of the DV is high. Furthermore, it was possible to observe moments when one DV was divided into two or when yeast inside the DV budded one cell at a time into the cytoplasm of alga-free *P. bursaria*. Although the DV membrane behavior when symbiotic *Chlorella* sp. is incorporated must be observed, the ability of *P. bursaria* to establish intracellular symbiosis with *Chlorella* sp. may be due to the dynamic differentiation of the DV membrane during phagocytosis.

## Supplementary Information

Below is the link to the electronic supplementary material.

Supplementary file1 This video shows the event of a single yeast cell budding from a DV containing multiple yeasts. Figure 5 was generated by cropping a portion of the video. Following the budding process, a single yeast cell was transported through the cytoplasm via cytoplasmic streaming of alga-free *P. bursaria, *moving away from the original DV (MP4 6061 KB)

Supplementary file 2 This video shows a DV containing multiple yeasts separating into two DVs. Figure 6 was created by cropping a portion of the video. The anterior side of *P. bursaria *was underside in the original video. The separation of the DV occurred as it encountered the cytopharynx of *P. bursaria* during cytoplasmic streaming, which likely triggered the separation due to the cytopharynx is a rigid structure covered with numerous cilia. Following the DV separation, each DV began to flow independently (MP4 7406 KB)

## Data Availability

The datasets used in this study are available from corresponding authors upon request.
